# Self-Regulation of Blood Oxygenation Level Dependent Response: Primary Effect or Epiphenomenon?

**DOI:** 10.3389/fnins.2016.00117

**Published:** 2016-03-24

**Authors:** Andrea Caria

**Affiliations:** ^1^Department of Psychology and Cognitive Science, University of TrentoRovereto, Italy; ^2^Institute of Medical Psychology and Behavioral Neurobiology, Eberhard-Karls-University of TübingenTübingen, Germany

**Keywords:** real-time fMRI, BOLD signal, neurofeedback, self-regulation

In the last decade, several studies indicated that neuronal activity can be volitionally modulated using real-time fMRI (rtfMRI) based neurofeedback. Human participants through rtfMRI paradigms can learn to regulate the blood oxygenation level dependent (BOLD) response in several localized cortical and subcortical regions (for extended reviews see Caria et al., 2012; Weiskopf, 2012; Sulzer et al., 2013). Increasing evidence also indicated that strengthening or weakening specific BOLD activity using rtfMRI training leads to significant changes in cognitive, emotional and motor behavior (Caria et al., 2012; Weiskopf, 2012; Sulzer et al., 2013). These findings suggested that rtfMRI might represent an important novel approach in cognitive neuroscience by potentially providing indications of cause-and-effect relationships between brain and behavior, and also in clinical applications (Subramanian et al., 2011; Linden et al., 2012; Ruiz et al., 2013; Sitaram et al., 2014).

Although rtfMRI studies extend and enrich a large body of literature demonstrating operant learning of neuronal oscillations, skepticism still exists on the validity and reliability of studies showing learned control of the BOLD response. In particular, it is still debated whether this phenomenon is a primary effect of learning or it just an epiphenomenon resulting, for instance, from repeated execution of some mental processes.

Here, I will discuss specific psychophysiological and neurophysiological mechanisms presumably underlying learned regulation of the BOLD response to attempt to clarify its nature.

## Operant learning of the BOLD response

Direct manipulation of brain activity grounds on the principles of operant conditioning that describe the relationship between changes in behavior (in this case the neurophysiological response) as consequence of contingent environmental events. Reinforcement is the mechanism that allows participants to increase the frequency of specific brain activity and to shape the desired pattern of neuronal activations. During operant learning of the BOLD response participants are reinforced proportionally to how much the ongoing metabolic signal approach or resemble the target level of activation in single or multiple regions of interest. The information of the ongoing signal fluctuations, provided as explicit visual feedback to the participants, represents the intrinsic reward.

Operant learning is an important approach for manipulating brain functions (Birbaumer and Kimmel, 1979) acting on intrinsic physiological properties of neuronal activity, which have been extensively shown to undergo classical and operant conditioning (Kandel and Schwartz, 1982).

The application of operant control principles to metabolic signals builds on several previous experiments demonstrating operant control of neuroeletric activity in non-human and human animals (Birbaumer and Kimmel, 1979; Fetz, 2007). Studies on volitional control of neural activity dates back to the late sixties and seventies when operant conditioning of central nervous system activity was demonstrated (Olds, 1965; Fetz, 1969; Shinkman et al., 1974). CNS unit conditioning was shown by operantly rewarding rats to increase the activity of midbrain neurons using intracranial stimulation (Olds, 1965). In 1969, Fetz demonstrated conditioning of the activity of single neurons in precentral cortex in anesthetized monkeys by reinforcing high rates of neuronal discharge with food reward and auditory or visual feedback of unit firing rates (Fetz, 1969). More recently, Brain-Computer Interface and Brain-Machine interface studies reported volitional control of cortical cell activity, in particular in the motor cortex, in both animals and humans (Serruya et al., 2002; Taylor et al., 2002; Carmena et al., 2003; Moritz et al., 2008), with food reward and visual feedback of neuronal ensembles' activity. Altogether these findings robustly proved that neuronal activity from localized brain areas can be manipulated through operant conditioning.

On the other hand, there are unclear aspects of operant conditioning of the BOLD response in humans that make rtfMRI neurofeedback paradigms not fully established. Among these is the role of mental and cognitive processes for learning. Some rtfMRI studies indicated that successful BOLD regulation relies on the use of mental imagery (Chiew et al., 2012; Banca et al., 2015; Blefari et al., 2015). In particular, these studies suggested that strategies based on mental imagery are important for learning BOLD control considering the partial common substrates for internal representations and overt behavior, which holds in particular for the primary motor (Jeannerod, 1995; Roth et al., 1996; Jeannerod and Frak, 1999; Niyazov et al., 2005) and visual areas (Kosslyn and Thompson, 2003; Klein et al., 2004; Slotnick et al., 2005). On the contrary, other studies indicated that a combination of cognitive strategies (mental imagery) and feedback information is critical for participants to learn BOLD regulation, and that cognitive and imagery alone is not sufficient (decharms et al., 2005; Bray et al., 2007; Caria et al., 2010; Scharnowski et al., 2012). In particular, mere repetition of mental strategies leads to an initial increase of BOLD signal but to a rapid decrease in the following consecutive runs (Caria et al., 2010). In fact, fMRI adaptation paradigms (Kourtzi and Kanwisher, 2001) show that task repetition leads to a decrease of the BOLD signal.

In addition, while it is true that imagery and cognitive processing certainly affect learning and physiological regulation, studies on operant control of brain oscillations suggest that feedback is more important than instructions for successful slow cortical potentials regulation (Roberts et al., 1989; Birbaumer et al., 1990). There are indications from EEG based neurofeedback studies that mental imagery is efficacious initially but it would be then dropped when participants become more confident with self-regulation (Leuthardt et al., 2004). Ultimately, control of neuroelectric activity turns to a highly implicit process over training, and the contribution of higher order cognitive processes becomes negligible. Indeed, control of neurophysiological signals can even be attained when participants have little or no direct experience of it (Rockstroh et al., 1984). However, until now there exist no clear demonstrations that these mechanisms, although plausible, hold also for learned metabolic signals.

In most rtfMRI studies participants were aware of the responses leading to reward. Only one study reported to have employed implicit visual task and feedback to learn BOLD control in V1/V2 areas, however participants still reported to have used explicit mental strategies (Shibata et al., 2011).

In short, the fact that operant control of brain activity, even single cell responses is possible also in animals speaks against a fundamental influence of cognitive factors on the effect of operant brain regulation. On the other hand, it is conceivable that in human participants the combination of specific conscious processes and operant strategies might support retention of learned BOLD control out of the laboratory setting, and might facilitate the (re-)activation of impaired or dormant mechanisms, in particular in patients.

## What neurophysiological mechanisms mediate learned BOLD control?

A further issue of rtfMRI experiments is the unclear neurophysiology of the BOLD signal, which prevents us to univocally interpret the behavioral changes induced by operant learning of the metabolic signal. We know from previous investigations on instrumental learning of neuroelectric signals that the brain has intrinsic neurophysiological processes allowing regulation of neural activity even in the absence of external stimuli (Wolpaw et al., 2002; Birbaumer and Cohen, 2007; Fetz, 2007). While no specific receptors directly support regulation of brain oscillations, it exists a complex neurovascular system that regulates and controls the BOLD signal.

At least three main blood flow regulatory mechanisms have been described: cerebral autoregulation—the brain ability to maintain a constant flow through changes of cerebral perfusion pressure—neurogenic regulation—the cerebral blood flow modulation through extensive arborization of perivascular nerves—and flow-metabolism coupling, or functional hyperemia—the brain capacity to vary blood flow to match metabolic activity (Peterson et al., 2011). Endothelial cells and astrocytes play a central role in all these regulatory mechanisms (Iadecola and Nedergaard, 2007): the former by providing several vasoactive factors for the regulation of cerebral blood flow and the latter because of their anatomical position that physically links the cerebral microvasculature with synapses.

Learned increase and decrease of BOLD activity might indeed be associated with variations in the flow-metabolism coupling. Local changes in neural activity have been shown to influence BOLD signal (Buxton et al., 2004; Logothetis and Wandell, 2004; Lee et al., 2010) through variations in cerebral blood flow (CBF), cerebral blood volume (CBV) and cerebral metabolic rate of oxygen consumption (CMRO2). It has been also demonstrated that changes in the coupling ratio of CBF and CMRO2 in response to neural activity strongly affect the BOLD response (Kim and Ogawa, 2012; Buxton et al., 2014). Considering such complexity of the neurovascular system, it is thus conceivable that learned control of the BOLD response might be based on these sophisticated regulatory mechanisms.

An intriguing perspective for rtfMRI neurofeedback arises from the hypothesis proposing that modulation of brain activity is directly and causally affected by changes of metabolic signals (Moore and Cao, 2008). Specifically, it has been conjectured that the brain vascular system, being a complex and interconnected network under tight regulatory control that occurs in close communication with neurons and glia, might directly contribute to information processing (Moore and Cao, 2008). Accordingly, the hemodynamic processes would not only support metabolic demand but also directly shape brain functions; self-regulation of metabolic signals might thus induce changes of neuronal activations. In line with this perspective non-invasive direct electrical stimulation (e.g., using transcranial direct electric stimulation, tDCS) targeting cerebral microvessels has been proposed to enhance brain functions trough changes of cerebrovascular function (Dutta, 2015; Pulgar, 2015).

So far, despite the increasing number of hypotheses and studies aiming to clarify the nature of the BOLD response, the neurometabolic coupling of this signal remains unclear. It has been shown that the BOLD fMRI response correlates with local field potentials (Logothetis et al., 2001; Viswanathan and Freeman, 2007), with spiking activity (Heeger and Ress, 2002), and with both LFP and spiking activity (Mukamel et al., 2005). In addition, BOLD signal can reflect different brain processing such as excitatory and inhibitory activity, neuromodulation, and bottom-up and top-down signals (Viswanathan and Freeman, 2007; Logothetis, 2008; Lee et al., 2010).

On the basis of this evidence it is difficult to infer whether the net effect of self-regulation of BOLD signal is excitatory, inhibitory or a combination or both. On the other hand, rtfMRI studies, although sometimes controversial, often reported an improvement of subjects' performance associated with increased amplitude of the BOLD response (decharms et al., 2005; Bray et al., 2007; Rota et al., 2009; Caria et al., 2010; Scharnowski et al., 2012, 2015; Zhang et al., 2013; Blefari et al., 2015). For instance, studies showed a decrease in reaction times for task execution associated with increase activity of the primary motor cortex (M1), and for task initiation after up-regulation of the SMA (Scharnowski et al., 2015). M1 activity was also positively correlated with accuracy improvement of performance during an isometric pinching task (Blefari et al., 2015) In the visual domain, increased visual perception was induced by increased BOLD response in the visual cortex (Scharnowski et al., 2012). Moreover, enhanced emotional response was associated with increased percentage BOLD signal change in the left anterior insula (Caria et al., 2010). Importantly, these studies reported poorer performance either during initial regulation runs or after down-regulation. Altogether these findings suggest that learned increase of the BOLD response might be associated with increased excitatory activity. This assumption would be also supported by studies showing improved memory performance associated with decreased BOLD activity in the parahippocampal cortex (Yoo et al., 2012; Scharnowski et al., 2015), which was then interpreted as result of increased brain resources for memory encoding because of decreased cortical processing (Yoo et al., 2012). Indeed, increased BOLD signal might occur from increased net excitation associated with glutamatergic activity (Logothetis, 2008). However, this represents only one possible interpretation, as larger BOLD signal can also be induced by concurrent increase of both glutamatergic and GABAergic activity, with changes in hemodynamic responses reflecting balanced local recurrent activity (Logothetis, 2008). In line with this alternative mechanism, it has been shown that enhanced perceptual sensitivity induced by fMRI feedback training results from a mixture of positive and negative activated voxels in the targeted regions of interest rather than from uniform positive or negative activity (Shibata et al., 2011).

Therefore, fMRI neurofeedback might also rely on, and possibly alter, the capacity of the cerebral cortex to generate persistent activity in the absence of sensory stimulation (Haider et al., 2006), which would in turn influence neuronal responsiveness to a wide range of inputs (Shu et al., 2003a). In addition, as the ongoing network activity can be turned on and off by synaptic inputs via electrical stimulation (Shu et al., 2003b), it can also be postulated its self-regulation via operant conditioning.

## Conclusions

In short, the observed behavioral changes induced by rtfMRI training might be related to several plausible neurophysiological processes, and it is still not possible to conclusively ascertain the actual mechanism on the basis of the current results. Multimodal measurements such as simultaneous EEG-rtfMRI acquisition, as well as alternative real-time functional imaging methods such as MR perfusion and MR spectroscopy, might help to clarify the neurophysiological mechanisms underlying learned control of the BOLD response. Nevertheless, there exist clear indications that self-regulation of BOLD signal is not an epiphenomenon but a primary effect of operant learning, which can rely on precise neurovascular regulatory mechanisms.

## Author contributions

The author confirms being the sole contributor of this work and approved it for publication.

### Conflict of interest statement

The author declares that the research was conducted in the absence of any commercial or financial relationships that could be construed as a potential conflict of interest.
